# Large neutral amino acid status in association with P:T ratio and diet in adult and pediatric patients with phenylketonuria

**DOI:** 10.1002/jmd2.12076

**Published:** 2019-09-16

**Authors:** Teresa D. Douglas, Anita M. Nucci, Ann M. Berry, Sarah T. Henes, Rani H. Singh

**Affiliations:** ^1^ Department of Human Genetics Emory University Atlanta Georgia; ^2^ Department of Nutrition Georgia State University Atlanta Georgia

**Keywords:** amino acids, dietary protein, nutrition, phenylketonuria, supplementation

## Abstract

**Background:**

Intake of large neutral amino acids (LNAA) may inhibit phenylalanine (PHE) transport across the blood brain barrier and assist with blood PHE control in patients with phenylketonuria (PKU). We evaluated the interrelationship between LNAA in plasma and diet on Phe:Tyr (P:T) ratio in patients with PKU and the influence of dietary factors on plasma LNAA markers.

**Methods:**

Plasma amino acid values and 3‐day food record analysis from two studies (34 male/30 female; age 4.6‐47 years) were examined. For pediatrics (<18 years) and adults (≥18 years) the relationship between P:T ratio, plasma LNAA, and dietary intake patterns were investigated.

**Results:**

Dietary factors influencing P:T ratio included intake of total protein (g/kg), medical food (MF) protein (g/kg, % below Rx), and LNAA (g) in the full cohort (*P* < .05). Associations were found between plasma valine and other dietary and plasma LNAA in pediatrics (*P* < .05) and plasma LNAA with dietary LNAA intake in adults (*P* = .019). Plasma P:T ratio was inversely associated with plasma LNAA concentrations in both age groups (*P* < .05). Aside from histidine in pediatrics (*P* = .024), plasma LNAA did not differ by having plasma PHE levels within or above the therapeutic range (120‐360 μmol/L). Plasma LNAA in both age groups was similar to reported healthy control values.

**Conclusion:**

P:T ratio is significantly tied to dietary LNAA, adherence to MF Rx, and plasma LNAA concentrations. Additionally, P:T ratio and valine may be effective clinical proxies for determining LNAA metabolic balance and LNAA quality of the diet in patients with PKU.

**SYNOPSIS**

Dietary factors play a role in LNAA plasma concentrations for the potential benefit of PHE control in the blood and across the blood brain barrier, while plasma valine and P:T ratio may serve as effective biomarkers of overall LNAA status.

## 1 INTRODUCTION

Phenylketonuria (PKU) is an autosomal recessive genetic disorder in which the enzyme, phenylalanine hydroxylase (PAH), produced in the liver, is deficient. The reduced activity of PAH causes phenylalanine (PHE) to build up in the blood and body tissues which is toxic to the central nervous system, most notably the brain.^1^ PKU is traditionally treated with a medical diet consisting of strict low amounts of dietary PHE and a synthetic, PHE free, amino acid‐based nutritional formula (medical food) that serves as the major dietary protein source.^1^, ^2^ The goal of the diet is to keep plasma blood PHE within the therapeutic range of 120‐360 μmol/L, the level needed for normal development and cognitive function across all ages.

Plasma concentration of large neutral amino acids (LNAA) may also play an important role in the treatment of PKU, particularly with the presence of LNAA in prescription medical foods and with prescribed LNAA supplements becoming more prevalent as a treatment option.^3^, ^4^ Large neutral amino acids include the essential amino acids histidine, isoleucine, leucine, methionine, threonine, tryptophan, phenylalanine, and valine as well as the nonessential amino acid tyrosine. Consumption of supraphysiologic LNAA supplements as a part of treatment in PKU may increase blood LNAA levels and reduce PHE concentrations in the blood and brain.^5^, ^6^ However, significance in PHE reduction only occurred in the absence of medical food. This highlights the importance of amino acid rich medical food in PHE control. Several studies have found clear associations between PHE control in PKU and medical food compliance, dosing, and amino acid content.^7^, ^8^, ^9^, ^10^ However, investigation of medical food LNAA content on P:T ratio is not represented in the literature. As long term P:T ratio has been found to associate with executive functioning, anxiety, and depression risk outcomes in PKU,^11^, ^12^ it is of clinical importance to determine the role of dietary LNAA from medical food and intact food sources on P:T ratio.

Mechanism of action regarding LNAA influence on metabolic control in PKU and brain function most likely involves competitive uptake inhibition in the gut and across the blood brain barrier (BBB).^13^, ^14^ L‐type amino acid transporter 1 (LAT1) is a common transport system for PHE and other LNAA across cellular membrane barriers, including the BBB.^5^ Higher concentrations of LNAA may inhibit excess PHE uptake at LAT1 sites.^15^ Supportive mouse studies demonstrate that LNAA substantially decrease brain PHE, reduce LNAA brain deficiencies, and lead to improved monoamine neurotransmitter status.^14^, ^15^, ^16^ However, as with LNAA and P:T ratio, the association between LNAA intake among patients with PKU on plasma LNAA concentrations has not been well‐established. Given the potential interaction that plasma LNAA and P:T ratio may have, this is important to know.

Therefore, the purpose of this study is to examine the effect LNAA intake from medical food and intact protein have on both P:T ratio and plasma LNAA in children and adults with PKU. These data are useful for determining intervention for optimizing LNAA status and P:T ratio in patients with PKU so as to improve clinical outcomes.

## 2 MATERIALS AND METHODS

### 2.1 Study population

**Figure 1:**
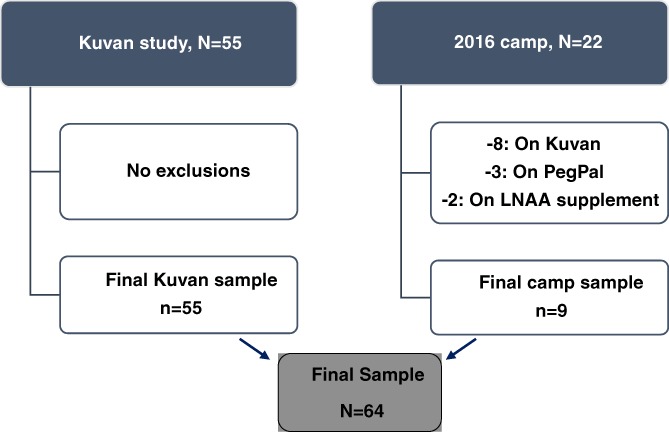
Flow chart of baseline study sample

The study sample (detailed in Figure 1) includes age diverse male and female participants from two previous studies by Emory University School of Medicine, Department of Human Genetics. Methods for these studies have been previously described in the peer‐reviewed literature.^17^, ^18^ For this analysis, the sample consisted of adolescent and adult females, within and outside of Georgia, who attended the Emory Metabolic Camp in the summer of 2016. Added to this sample was males and females 4 years of age and older from 2009 baseline data of PKU patients thereafter introduced to sapropterin (Kuvan, Biomarin, Inc.). Age appropriate informed consent and assent procedures were followed in all circumstances. All patients had been diagnosed with PKU through the Newborn Screening Protocol. If a study subject was present in both camp and sapropterin data sets, the subject was deleted from the older baseline sapropterin set and retained in the 2016 camp data set for analysis. One exception was a subject on sapropterin in the camp data set and thus retained in the 2009 baseline data set. Exclusion criteria were non‐PKU individuals, pregnant and nursing females, patients under the age of 4 years, those taking either a tyrosine or LNAA supplement, those receiving pharmacologic treatments for their PKU (Kuvan and PEGPAL) at time of data collection, and patients with cognitive deficits affecting ability to provide informed consent or follow study instructions.

### 2.2 Study design

This is a secondary retrospective cohort study. Deidentified data previously collected as part of the Institutional Review Board (IRB) approved Emory Metabolic Camp and sapropterin study protocols were used as described in the prior section. Each patient was assigned a random study identification number. Variables included demographic characteristics (age in years, gender, and race), anthropometrics (weight, height, calculated BMI), nutrient intake from dietary food records, plasma amino acids, and determinants of dietary compliance. Ethics approval for this secondary retrospective study was received from the Georgia State University IRB and met Emory University IRB standards.

### 2.3 Anthropometric and dietary measures

Weight in kilograms (kg) was measured with a standing scale. Height in centimeters (cm) was determined using a stadiometer. Body mass index (BMI) was calculated [weight (kg) / height (meters)^2^]. Subjects under 18 years have BMI reported as a percentile. All measures were conducted by trained research staff. Participants at the Emory Metabolic Camp and at the baseline visit for the sapropterin study completed a 3‐day food record prior to their visit. A registered dietitian (RD) from Emory University analyzed collected food records for nutrient intake using Nutrition Data System for Research (NDSR; Nutrition Coordinating Center, University of Minnesota, 2016). Participants were determined to be adherent with their PKU diet if blood PHE levels were within recommended range (120‐360 μmol/L).^19^ To determine adherence to prescribed medical food (MF), reported intake was subtracted from prescribed and converted to the percent intake below prescription (Rx). For adherence to dietary PHE, prescribed dietary PHE was subtracted from reported PHE intake and converted to percent intake above Rx.

### 2.4 Blood sampling

Green top sodium heparin vacutainers were used for blood sampling. Plasma amino acids were analyzed using quantitative ion exchange chromatography at the Emory Genetics Laboratory. Plasma LNAA reported, aside from PHE and Tyr, included valine, leucine, isoleucine, methionine, threonine, and histidine. For a subcohort in the study (n = 50) tryptophan was also reported. Sum plasma LNAA was calculated by adding the individual plasma LNAA concentrations, a method previously utilized in the literature.^20^ Plasma tryptophan for the sum LNAA was not included in the complete cohort analysis but was calculated in for the subcohort. The same approach was taken for dietary tryptophan when determining sum LNAA in the diet. In the case of sum LNAA associations with P:T ratio, tyrosine was removed from the sum to reduce confounding. Plasma PHE was excluded from sums to prevent confounding by high PHE levels, and as PHE control was already being evaluated.

### 2.5 Statistical analysis

Frequency analysis was used to describe demographic, anthropometric, nutrient intake, and clinical characteristics of the combined sample. Normality testing was conducted on all continuous variables to determine whether parametric or nonparametric statistics should be used. Correlations were used to identify potential cofactors such as age, gender, and dietary compliance. To control for possible age related differences relevant to dietary and metabolic parameters, the sample was divided into pediatrics (<18 years) and adults (≥18 years). Kendall's tau and multivariate regression were used to assess relationships between plasma LNAA, plasma P:T ratio, LNAA intake from diet sources and in association with adherence to MF Rx. The *t* test comparisons were used to detect potential group differences between sum (plasma and intake) LNAA values with and without tryptophan. Compliance status of the study sample was determined by plasma PHE concentrations. Those with plasma PHE exceeding 360 μmol/L were categorized as “noncompliant” with their PKU treatment plan independent of reported intakes on their diet record Differences based on PKU diet compliance were investigated with Mann‐Whitney *U* test or *t* test. Margins of error are reported as 25th and 75th percentiles or SD dependent on normality of the data. Multiple comparisons due to separation of sample by age group were Bonferroni adjusted. All statistical analyses were performed with SPSS (version 24.0, SPSS Inc. Chicago, IL). IBM SPSS Sample Power (version 3) was used to assess needed sample size for a regression model with up to three variables at a power of 0.80. Alpha was set at 0.05.

## 3 RESULTS

### 3.1 Demographic, anthropometric, and nutrient intake characteristics

The final cohort included 64 participants (53% male) with an age range of 4.6‐47 years. The majority of participants were in the sapropterin study (n = 55) while the remaining (n = 9) attended the Emory Metabolic Camp in the summer of 2016 (Figure 1). Total study power based on sample size achieved is 0.99, with a minimum of 29 needed for a power of 0.80. The demographic, anthropometric, and prescribed diet adherence characteristics of the study group are shown in Table 1. Mean (±SD) per kilogram protein intake for the complete study group was 1.30 g/kg (±0.64). After subdivision by protein source, whole group mean (±SD) dietary intact protein intake was 0.41 (±0.32) g/kg and 0.89 (± 0.62) g/kg for MF protein intake. Of the 61 participants on MF, one was prescribed a glycomacropeptide (GMP) product, while the others were on a PHE‐free synthetic amino acid MF. Among those on prescribed MF, 43 (70%) reported consuming full prescribed amount. The median percent dietary protein from MF in the full cohort was 73.9% (IQR; 51.8, 84.1). Of 59 participants with reported dietary PHE restriction, 81% (n = 52) had intake at least 5%, above recommended, with 43 subjects exceeding PHE intake recommendation by more than 20%. Per expectations, nonadherence to dietary PHE restriction was greater in adults, with median PHE intake 61.3% above recommended. In pediatrics, median excess PHE intake was 26.3% above recommended.

**Table 1:** Baseline characteristics of the study cohort

Characteristic	N = 64	Mean ± SD
Gender, n		
Male:	n = 34	
Female	n = 30	
Age group (n)^a^	Pediatric (n = 37)	10.4 ± 4.2
	Adult (n = 27)	27.9 ± 8.6
BMI, percentile	Pediatric	60.2 ± 29.7
BMI (kg/m^2^)	Adults	30.7 ± 8.3
		n (%)
Phe intake above recommended (out of n = 59)^a^	Pediatric (n = 34)	27 (79.4%)
	Adults (n = 25)	20 (80.0%)
MF intake below Rx (n = 64)	Pediatric (n = 37)	10 (27.0%)
	Adults (n = 27)	10 (48.0%)

*Note*. Pediatric: <18 years of age. Adults: ≥18 years of age.

Abbreviations: BMI, body mass index; MF, medical food; Phe, phenylalanine; Rx, prescription; SD, standard deviation.

a For three subjects, medically recommended Phe intake was not available due to length of time off diet. Two subjects had mild hyperphe and thus not Phe restricted.

Comparisons of dietary sum LNAA (g) with and without tryptophan, and plasma sum LNAA (μmol/L) with and without tryptophan yielded no significant differences (*P* = .54, *P* = .14; respectively), validating outcomes in the subcohort in which plasma tryptophan concentrations were included.

### 3.2 Dietary factors affecting plasma P:T ratio

In the separate age groups, dietary LNAA (g) were not associated with P:T ratio. For pediatrics, intake of protein (g, g/kg), energy (kcal, kcal/kg), and ratio of MF to intact protein were also not associated with plasma P:T ratio. In adults, g/kg intake of total protein and MF protein were each inversely correlated with P:T ratio (τ = −0.442, *P* = .001) (τ = −0.337, *P* = .014), emphasizing the importance of dietary adherence on metabolic control. Moreover, the percentage of MF protein the patient consumed below their prescription had a direct correlation with P:T ratio (τ = 0.307, *P* = .029), though not after Bonferroni correction.

**Figure 2:**
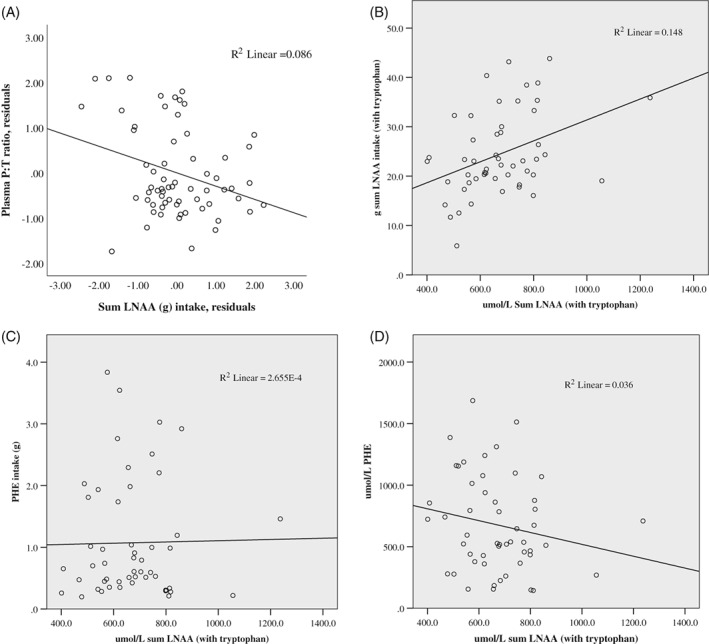
Correlations within full cohort (tryptophan included in sum of LNAA)–(A) P:T ratio with dietary sum LNAA intake (partial correlation with residuals)*,^21^ (B) plasma sum LNAA with dietary sum LNAA, (C) plasma sum LNAA with dietary PHE intake, (D) Plasma PHE with plasma sum LNAA. *P* values are for partial correlations controlling for age

Within the full cohort, when controlled for linear age, P:T ratio was inversely associated with dietary intake (g) of the LNAA sum (Figure 2A), and with intake of specific LNAA (*P* ≤ .048) other than threonine (*r* = −0.224, *P* = .054), tryptophan (*r* = −0.241, *P* = .057), and PHE (*r* = −0.004, *P* = .978). The full cohort, as with the adult subcohort, also demonstrated strong association of P:T ratio with MF intake (g/kg, % below Rx) (*r* = −0.273 *P* = .030; *r* = 0.433 *P* < .001) and total protein intake (g, g/kg) (*r* = −0.273 *P* = .031; *r* = −0.337 *P* = .007).

### 3.3 Dietary factors affecting plasma LNAA

Evaluating correlations between individual dietary LNAA intake (g) and plasma LNAA levels, significant positive associations were found in the full cohort with the sum LNAA values (Figure 2B) and in the pediatric subcohort with isoleucine (τ = 0.25, *P* = .029) and valine (τ = 0.35, *P* = .003). In adults, positive associations were found with valine (τ = 0.29, *P* = .033) and with sum LNAA, (τ = 0.39, *P* = .019). Dietary tyr correlated with plasma valine for both pediatrics (τ = 0.319, *P* = .006) and adults (τ = 0.274, *P* = .045) along with sum plasma LNAA (*P* ≤ .044). Dietary tyr for pediatrics also correlated with plasma isoleucine (τ = 0.245, *P* = .035) and tryp (τ = 0.276, *P* = .027). Once Bonferroni corrected, the only significant correlations were with valine in the pediatric group, and between dietary sum LNAA and plasma sum LNAA (with tryptophan) in the adults.

Of note, in the pediatric cohort, plasma valine had strong positive correlation with intake of all dietary LNAA (*P* ≤ .015) excepting PHE and tryptophan, and with plasma LNAA (*P* ≤ .019), excepting PHE. In adults, valine likewise directly associated with the plasma BCAA (*P* ≤ .002), threonine (τ = 0.284, *P* = .039), and the sum plasma LNAA (τ = 0.0742, *P* < .001). Only dietary threonine (g) (τ = 0.331, *P* = .016) and tryptophan (g) (τ = 0.342, *P* = .012) intakes correlated with plasma valine in adults once Bonferroni corrected.

Plasma histidine correlated with MF protein (g) intake in pediatrics (τ = 0.33, *P* = .004) and total protein intake (g/kg) in adults (τ = 0.39, *P* = .005). Histidine associations held after Bonferroni correction. No significant correlations were found between plasma LNAA and intake measures of dietary PHE (Figure 2C), medical food protein, intact protein, total protein, or sex in either age group. Comparison of LNAA markers by compliance status (as determined by plasma PHE above or below 360 μmol/L) across age groups were not statistically significant, with exception of significantly higher plasma histidine (*P* = .024, Bonferroni adjusted) in dietary compliant vs noncompliant pediatrics (mean ± SD: 80.7 ± 13.4 vs 69.0 ± 12.6 μmol/L). Even so, histidine intake (g/day) was similar across both pediatric compliance groups (medians: 1.36 vs 1.46 g/d; *P* = 0.376).

### 3.4 LNAA interaction with plasma PHE, tyr, and P:T ratio

In the full cohort controlled for age, plasma PHE associated inversely with the sum plasma LNAA (Figure 2D), and with individual LNAA results (*P* ≤ .027) except for valine, isoleucine, and leucine which were not statistically significant. Plasma tyr had significant positive associations with LNAA outcomes (*P* ≤ .028) other than plasma methionine (*r* = 0.246, *P* = .052) and histidine (*r* = 0.241, *P* = .058). Median plasma P:T values for adults was 16.2 (IQR: 8.9, 34.0) and 9.8 (IQR: 5.5, 20.3) for pediatrics. For both pediatric and adult groups with PKU, plasma P:T ratio had significant inverse associations with plasma threonine, histidine, and sum plasma LNAA (tyr removed) (Table 2). When evaluating the full cohort with Kendall's tau, P:T ratio had significant inverse correlations with all plasma LNAA concentrations (*P* ≤ .028) except for isoleucine (τ = −0.166, *P* = .056). When, controlling for linear age, isoleucine was also inversely correlated with P:T ratio (*r* = −0.334, *P* = .007).

**Table 2:** Association^a^ of P:T ratio with individual and sum plasma LNAA (excluding tyrosine)

Plasma outcome marker(μmol/L)	Pediatrics (τ)^b^(n = 37)	Adults (τ)^b^(n = 27)	Complete sample^c^(τ) (n = 64)
Histidine	–0.336**	–0.345*	–0.295**
Isoleucine	–0.240	–0.196	–0.166
Leucine	–0.220	–0.233	–0.189*
Methionine	–0.194	–0.251	–0.258**
Threonine	–0.281*	–0.475**	–0.344**
Tryptophan	–0.254 (n = 32)	–0.251 (n = 18)	–0.285** (n = 50)
Valine	–0.215	–0.291	–0.225*
Sum LNAA (w/o tryp and tyr)	–0.293*	–0.459**	–0.559**
Sum LNAA (w/o tyr) (n = 50)	–0.299* (n = 32)	–0.345 (n = 18)	–0.527** (n = 50)

Abbreviation: LNAA, large neutral amino acids.

* *P* < .05.

** *P* < .01.

^a^ Kendall‐tau.

^b^ Bonferroni adjusted for separate age groups.

^c^ Significant associations of complete cohort confirmed with multivariate regression adjusting for linear age.

### 3.5 Plasma LNAA and LNAA intake compared to general population

There is no general population standard, or matched non‐PKU cohort, to compare against the mean plasma LNAA concentrations in our study group. Nevertheless, a study conducted by Scriver et al^22^ examined plasma amino acid levels in healthy adults and another published by Lepage et al^21^ reported proper methods of obtaining plasma amino acid levels in a healthy pediatric population. Based on these two studies, our cohort had plasma levels of LNAA comparable to that observed in healthy children and adults, with the exception of PHE (Table 3A,B).

**Table 3:** P:T and LNAA intake and plasma values (μmol/L) in PKU cohort compared to published values for healthy pediatric (A) and healthy adult (B) groups

LNAA	N	Intake (mg/day) Median, IQR	Plasma (μmol/L) Mean, SD	Lepage et al 1997^a^ (μmol/L)^21^ Median	Plasma RR^b^ (μmol/L)
P:T	37	NA	13.62 ± 11.51	‐	‐
Phenylalanine	37	473 (303,779)	551 ± 298	49,57	26‐91
Tyrosine	37	4239 (3424,5666)	52 ± 19	55,65	24‐115
Tryptophan	37/31	667 (524,841)	46 ± 14	56,74	‐
Threonine	37	2364 (2000,2878)	108 ± 31	94,131	35‐226
Isoleucine	37	3094 (2616,4107)	50 ± 14	54,60	22‐107
Leucine	37	5377 (4490,6527)	101 ± 28	110,128	49‐216
Methionine	37	1106 (887,1305)	22 ± 5	20,26	7‐47
Valine	37	3600 (3060,4677)	206 ± 55	199,233	74‐321
Histidine	37	1451 (1167,1678)	73 ± 14	78,92	41‐125
Sum LNAA (without Tryptophan)	37	21 218 (18000,26365)	611 ± 126	‐	‐
Sum LNAA (with tryptophan)	37/31	22051 (18543,27102)	661 ± 138	‐	‐
**LNAA**	**N**	**Intake (mg/day)Median, IQR**	**Plasma (μmol/L)Mean, SD**	**Scriver et al 1985** c **(μmol/L)** 20 **Mean**	**Plasma RR** b **(μmol/L)**
P:T	27	NA	20.6 ± 13.5	‐	‐
Phenylalanine	27	997 (484,2030)	929 ± 467	58	49‐76
Tyrosine	27	4771 (2745,7043)	52 ± 20	64	41‐78
Tryptophan	27/17	1027 (715,1595)	40 ± 14	‐	‐
Threonine	27	3005 (2446,4054)	117 ± 57	145	85‐186
Isoleucine	27	3300 (2690,4521)	51 ± 12	64	40‐83
Leucine	27	6255 (4626,8107)	107 ± 26	133	88‐158
Methionine	27	1474 (1162,1614)	20 ± 4	24	21‐41
Valine	27	4565 (3283,5370)	218 ± 79	264	164‐275
Histidine	27	1896 (1586,2548)	75 ± 15	94	52‐128
Sum LNAA (without Tryptophan)	27	26125 (19536,33716)	639 ± 166	‐	‐
Sum LNAA (w tryptophan)	27/17	26840 (20324,35355)	692 ± 178	‐	‐

Abbreviations: IQR, interquartile range; LNAA, large neutral amino acids; NA, not applicable; P:T, Phenylalanine:Tyrosine ratio; SD, standard deviation; mg, milligrams; μmol, micromoles; L, liter.

a Healthy pediatric controls. Reported medians for 6 years and 16 years, respectively. Dietary data not collected by authors.

b Healthy population reference range (RR) as reported by Emory Genetics Laboratory.

c Healthy adult controls. Dietary data not collected by authors.

## 4 DISCUSSION

This study adds to the research literature on LNAA and their role in managing the metabolic needs of those with PKU. As nutritional and pharmacologic therapies for PKU change, dietary patterns do as well, which can affect health outcomes in diverse ways. The general literature consensus is that LNAA supplementation or specific MF higher in LNAA could benefit patients with PKU, even more so combined with metabolic PHE control. Particularly worth noting are the LNAA supplements available now for PKU patients to be taken in addition to or as a replacement for traditional MF.^22^, ^25^ One study investigated the combination of sapropterin with LNAA supplementation, revealing a synergistic effect on serum melatonin in BH4 responders.^26^

Our study focused on plasma LNAA and LNAA intake within the limits of PKU dietary adherence. Interestingly, we found in our study an inverse association of P:T ratio with multiple LNAA and the sum of LNAA in plasma (Table 2) even though compliance status as determined by plasma PHE levels was minimal in its effect on LNAA. Therefore, poor maintenance of plasma P:T ratio may be of greater relevance to LNAA balance than plasma PHE alone, potentially attenuating the benefits of increased dietary LNAA. That LNAA other than PHE in the diet had a greater influence on both P:T ratio and LNAA concentrations suggests competitive transport between PHE and the LNAA at the gut level. Thus, greater LNAA in the diet, though not a substitute for PHE control, are effective when combined with a holistic approach to PKU treatment that promotes the right balance of P:T in the blood.

Although human research into the effects of LNAA intake on brain PHE is limited, one study observed a lowering of brain PHE levels with increased LNAA intake, as measured by Magnetic Resonance Spectroscopy. This occurred despite no observed increase in plasma LNAA concentrations.^5^ Schindeler et al reported blood isoleucine, threonine, tyrosine, valine, lysine, and histidine levels to be higher when subjects received LNAAs in their MF mixtures, although neither data nor level of significance for the reported relationship was provided in their manuscript. Our results, although showing a clear association between dietary LNAA intake and sum plasma LNAA, when we evaluated distinct LNAA, valine particularly stood out. The noted association of valine with both plasma and dietary LNAA, as well as P:T ratio, indicates valine may be an important marker of LNAA status, dietary adherence, and metabolic control in patients with PKU. Hence, there is potential for valine's clinical application as a proxy of LNAA balance in pediatrics with PKU, perhaps in combination with P:T ratio. Why specifically valine was significant as opposed to other LNAAs is unclear. However, given the role of valine in PHE access across the blood brain barrier, this finding is of importance. The limited associations between dietary LNAA and plasma concentrations may be due to homeostatic mechanisms as the amino acids are taken up by cells to be used and metabolized for functional purposes. For example, a clinical study found that threonine catabolism via oxidation increases during dietary threonine supplementation as either free amino acids or from intact protein,^27^ although increases in plasma threonine were still observed. A similar effect may explain the increase in plasma valine seen with higher valine in the diet. However, clinical measures of downstream LNAA metabolites (ie, respective keto‐acids, histamine from histidine, cysteine from methionine) in patients with PKU, combined with dietary intake and plasma concentrations, are needed to identify the mechanisms that maintain stable LNAA plasma levels relevant to diet patterns.^28^ Additionally, measuring flux across membrane barriers for cellular uptake could add further insight, particularly as studies have shown that prescribing LNAA supplements to patients with PKU may be an effective therapy^3^, ^15^, ^29^ due to competitive inhibition of PHE across both the gut and blood brain barriers.

Rohde et al^30^ examined LNAA status along with other nutrients in a PKU patient sample similar to our own study sample by evaluating the total protein intake in those who consumed medical food vs those who did not. The researchers found no significant difference in the total protein intake and no deficit in one LNAA intake, which is consistent with our overall findings. As expected, many participants in our study exceeded recommended intake of PHE and underconsumed their medical food compared to their Rx (Table 1).

Within our study, dietary influence regarding protein source, MF or intact protein and dietary compliance status as measured by plasma PHE, had little bearing on plasma LNAA concentrations, although extent of noncompliance as determined by plasma PHE was a factor in lower plasma histidine concentrations among pediatrics. Even so, most still fell within the clinical reference range of 50‐104 μmol/L (LabCorp) for histidine.

There are limitations within the current study. The majority of participants (75%) were noncompliant as evidenced by plasma PHE levels >360 μmol/L, which reduced the size of the compliant subgroup for our analysis. Unfortunately, high numbers of noncompliance with plasma PHE and diet requirements, particularly among adolescents and adults, are common in the PKU medical population.^31^, ^32^, ^33^, ^34^ Another limitation was that plasma tryptophan could not be calculated into the sum of plasma LNAA concentrations in 14 cases. This may have affected the sum of the plasma LNAA concentration values and the significance of associations in the analysis. However, subgroup analysis among participants with plasma tryptophan available was completed, and results were similar to the full analysis that excluded tryptophan.

## 5 CONCLUSION

Our research has demonstrated a clear inverse relationship between LNAA concentrations in the plasma and P:T ratio, with dietary and plasma tyrosine of greater weight than PHE. We also found that P:T ratio correlated with dietary intake of total protein, MF protein, and LNAA (in the full cohort, Figure 2A). In addition, associations were discovered with plasma valine when correlated with other plasma LNAA, dietary LNAA intake, in conjunction with P:T ratio. This strength of association valine had with multiple dietary and plasma markers was distinct among the other LNAA. The results support the premise that both P:T ratio and valine may be clinically valuable predictors and potential mediators of LNAA balance among individuals with PKU. More studies are needed to best apply LNAA nutrition either within the PKU standard diet or as supplemental therapy, while optimizing P:T balance and long‐term health in PKU. Such research would investigate specific mechanisms of interaction between diet, LNAA plasma concentrations, P:T ratio and downstream metabolism and utilization of LNAA, as well as the efficacy of valine and P:T ratio in predicting LNAA balance in PKU.

## CONFLICT OF INTEREST

The authors have no relevant financial relationships or conflicts of interest to disclose.

## DETAILS OF ETHICS APPROVAL

Ethical approval for the study was obtained from the Emory University Institutional Review Board for the Emory Metabolic Camp protocol (IRB # 2447) and the Sapropterin (Kuvan) Study (IRB #7828). Ethical approval was also received from the Georgia State University Institutional Review Board (H17510).

## PATIENT CONSENT STATEMENT

All patients who participated in the study and are represented in the data provided written informed consent and/or assent as age appropriate.

## DOCUMENTATION OF APPROVAL FOR CARE AND USE OF LABORATORY ANIMALS

N/A.

## AUTHOR CONTRIBUTIONS

T.D.D., A.M.N., A.M.B., S.T.H., and R.H.S. have studied the concept and design, original drafting of manuscript and follow up revisions, and critical review of the manuscript. A.M.B., A.M.N., T.D.D., and R.H.S. have prepared the interpretation of data. T.D.D., A.M.N., and A.M.B. prepared the statistical analysis.
